# Psychometric Properties and Validation of the Positive and Negative Suicide Ideation (PANSI) Inventory in an Outpatient Clinical Population in Malaysia

**DOI:** 10.3389/fpsyg.2015.01934

**Published:** 2015-12-21

**Authors:** Aishvarya Sinniah, Tian P. S. Oei, Karuthan Chinna, Shamsul A. Shah, T. Maniam, Ponnusamy Subramaniam

**Affiliations:** ^1^Department of Psychiatry, Universiti Kebangsaan Malaysia Medical CentreKuala Lumpur, Malaysia; ^2^School of Psychology, The University of QueenslandBrisbane, QLD, Australia; ^3^Department of Psychology, James Cook University SingaporeSingapore, Singapore; ^4^Department of Psychology, School of Social and Behavioural Sciences, Nanjing UniversityNanjing, China; ^5^Department of Social and Preventive Medicine, Julius Centre University of Malaya, University of MalayaKuala Lumpur, Malaysia; ^6^Department of Community Medicine, Universiti Kebangsaan Malaysia Medical CentreKuala Lumpur, Malaysia; ^7^Health Psychology Programme, Universiti Kebangsaan MalaysiaKuala Lumpur, Malaysia

**Keywords:** mental health, suicide ideation, confirmatory factor analysis, culture, Malaysia

## Abstract

The PANSI is a measure designed to assess the risk and protective factors related to suicidal behaviors. The present study evaluated the psychometric properties and factor structure of the Positive and Negative Suicide Ideation (PANSI) Inventory in a sample of clinical outpatients at a major hospital in Malaysia. In this study, 283 psychiatric patients and 200 medical (non-psychiatric) patients participated. All the patients completed the PANSI and seven other self-report instruments. Confirmative factor analysis supported the 2-factor oblique model. The internal consistency of the two subscales of PANSI-Negative and the PANSI-Positive were 0.93 and 0.84, respectively. In testing construct validity, PANSI showed sizable correlation with the other seven scales. Criterion validity was supported by scores on PANSI which differentiated psychiatric patients from medical patients. Logistic regression analyses showed PANSI can be used to classify the patients into suicidal or non-suicidal. The PANSI is a reliable and valid instrument to measure the severity of suicidal ideation among clinical outpatients in Malaysia.

## Introduction

Suicide is a major social problem worldwide. It is recognized as one of the three leading causes of death among those aged 15–44 years in worldwide (WHO, 2012). Attempted suicide is estimated to be 10–20 times more frequent than suicide (Beautrais and Mishara, 2007). In Malaysia, suicide rates have increased by 60% over the past 45 years. It is also estimated that seven people in Malaysia attempt suicide daily (Malaysian Psychiatric Association, 2007; Aishvarya et al., 2013).

The seriousness of this social problem has led to much research in the development of instruments to assess and measure suicide related behaviors. Some of the instruments include The Beck Scale for Suicide Ideation (BSI; Beck and Steer, 1991), The Suicide Behaviors Questionnaire (SBQ; Linehan and Addis, 1983), The Suicide Probability Scale (SPS; Cull and Gill, 1982), Suicidal Ideation Questionnaire (SIQ; Reynolds, 1987), Reasons For Living Inventory (RFL; Linehan et al., 1983), and Modified Scale for Suicidal Ideation (MSSI; Miller et al., 1986).

Osman et al. (1998) designed the Positive and Negative Suicide Ideation (PANSI) Inventory, a 14-item self-report instrument. PANSI was developed to assess the frequency of suicide ideation that incorporates both negative risk and protective factors among adolescents and adults aged ≥14 years. PANSI is composed of two factors: six items of positive ideation and eight items of negative ideation. Examples of positive ideation items are “Felt that you were in control of most situations in your life” and “Felt excited because you were doing well at school or at work” Examples of negative ideation items include “Felt so lonely or sad you wanted to kill yourself so that you could end your pain” and “Thought about killing yourself because you felt like a failure in life.” In PANSI, each item is rated on a 5-point Likert scale ranging for 1 (*none of the time*) to 5 (*most of the time)*. The PANSI items are rated based on the time reference of “the past 2 weeks, including today.”

Studies have been conducted in different samples investigating the psychometric properties of the PANSI. Osman et al. (1998) reported good internal consistency for PANSI-positive (α = 0.80) and PANSI-negative (α = 0.91), using a sample of 450 undergraduates. Confirmatory factor analysis showed that the two factor oblique model was the best fit. Lester (1998) reported that PANSI scores were useful in predicting suicidal ideation as Beck Hopelessness Scales scores when tested for its psychometric properties in a sample of 69 undergraduates. A two factor oblique model was found to be the best fitting model when tested in a sample of adolescent psychiatry inpatient (Osman et al., 2002), normal adolescents (Osman et al., 2003), and young adults (Muehlenkamp et al., 2005). The Chinese version of PANSI was tested among 2341 middle and high school students in Taiwan. Coefficient alpha for the PANSI was good with 0.94 for PANSI-negative and 0.86 for PANSI-positive, and the two factor oblique model was again found to be the best fit (Chang et al., 2009). Among the adolescent psychiatry inpatients, Cronbach's alpha was 0.96 for PANSI-negative and 0.89 for PANSI-positive. Meanwhile, among the normal adolescents, the reliability of PANSI was reported to be adequately high (alpha value > 0.70).

Although PANSI has been validated in clinical and non-clinical settings using subjects in a wide range of age groups in Western societies, it is still necessary to validate this instrument in an Eastern society. Malaysia consists of three major ethnic groups; Indian, Chinese and Malay. These ethnic groups have their own unique values and culture. To date no studies have been conducted among clinical outpatient in Malaysia to examine the factor structures and other psychometric properties of PANSI. Thus, our study was designed to (a) examine the factor structure and psychometric properties of PANSI in a sample of adult clinical outpatients in Malaysia (b) examine the reliability and validity of PANSI with other measures of suicide behavior and general psychopathology.

## Methods

### Instruments

#### Translating and back translating of PANSI

In this study, two bilingual psychiatry registrars and two clinical psychologists with Master's degrees translated and back translated the English version of PANSI and other instruments. A professional language interpreter was asked to proofread the translated questionnaires. This is to ensure the suitability and to resolve word ambiguity issues after the translation process. The back-translated versions were similar to the original versions and to each other. The minor differences in both languages were reconciled.

#### Other scales

##### Demographic questions

One question was developed specifically for this study to collect data on history of attempted suicide: “0” I have never attempted suicide, “1” I have attempted suicide once and “2” I have attempted suicide two or more times. Later, for data analysis purposes, those who attempted suicide either once or more than once were categorized as one group.

#### The depression anxiety stress scale-21

The Depression Anxiety Stress Scale-21 (DASS-21; Lovibond and Lovibond, 1995) is a brief measure of depression, anxiety, and stress, with seven items in each domain. DASS-21 has good reliability and validity across various setting (Anthony et al., 1998; Clara et al., 2001; Henry and Crawford, 2005; Gloster et al., 2008; Oei et al., 2013). Each DASS-II item is rated on a 4–point scale from 0 (did not apply at all) to 3 (applied to me very much, or most of the time). In the Malaysian general population, DASS-21 has shown very good Cronbach's alphas of 0.84, 0.74, and 0.79, respectively for depression, anxiety and stress subscales. The factor loading values for the items ranged between 0.39 and 0.73 (Ramli et al., 2007, 2009).

#### Reasons for living inventory (RFL)

The Reasons for Living Inventory (RFL) was developed by Linehan et al. (1983) to examine the cognitive factors associated with reasons to live despite hardship. The RFL was developed based on a cognitive-behavioral theory to examine the cognitive factors, which act as the buffer toward suicidal behavior. Various studies have reported satisfactory levels of reliability and validity for this scale (Cole, 1989; Osman et al., 1991, 1992, 1993, 1999; Gutierrez et al., 2002). The Reasons for Living Inventory (Linehan et al., 1983) has 48-items with specific reasons for an individual for not committing suicide. A total of six subscales were identified based on four separate factor analyses which were carried out on two samples of normal adult subjects: (1) suicidal and coping belief, (2) responsibility to family, (3) child-related concerns, (4) fear of suicide, (5) fear of social disapproval, and (6) moral objections. Each item in this inventory is rated at six levels of importance ranging from 1 (not at all important) to 6 (extremely important).

#### Beck hopelessness scale (BHS)

The Beck Hopelessness Scale (BHS; Beck et al., 1974) is a 20-item, self-report measure of the symptoms of hopelessness about the future. Psychometric investigations have shown satisfactory levels of reliability and validity for this scale in clinical and non-clinical samples (Beck et al., 1974; Steer et al., 1993; Glanz et al., 1995). Each item in this scale is rated as true or false and a total score is obtained by summing responses; higher scores are indicative of greater hopelessness. Examples of questions asked: “All I can see ahead of me is unpleasantness rather than pleasantness” and “My future seems dark to me.”

#### Provision of social relations (PSR)

The Provision of Social Relations (PSR; Turner et al., 1983) is a 15-item instrument designed to measure components of social support. The factor analysis for PSR revealed two dimensions of supports: (1) family support and (2) friend support. A total score can be obtained by summing the scores on the two dimensions. Higher scores reflect more social support.

#### Satisfaction with life scale (SWLS)

The 5-item Satisfaction With Life Scale (SWLS; Diener et al., 1985) is an instrument to assess life satisfaction. The SWSL reveals an individual's own judgment of his or her quality of life. Each item is responded to on a1–7 scale from “strongly disagree” to “strongly agree.” Item scores are summed for a total score, which ranges from 5 to 35, with higher scores reflecting more satisfaction with life. Previous studies have shown that the SWLS has high internal consistency with coefficient alpha ranging from 0.79 to 0.89 (Pavot and Diener, 1993).

#### Rosenberg self esteem scale (RSE)

Rosenberg Self Esteem Scale (RSE; Rosenberg, 1965) is a ten-item instrument with items answered on a four-point scale—from strongly agree to strongly disagree. The scale generally has high reliability: test-retest correlations are typically in the range of 0.82–0.88, and Cronbach's alpha for various samples are in the range of 0.77–0.88.

#### The adult trait hope scale (ATHS)

The Adult Trait Hope Scale (ATHS; Snyder et al., 1991) contains 12 items. Four items measure pathways thinking, four items measure agency thinking, and four items are fillers. Participants respond to each item using the 8-point scale ranging from definitely false to definitely true. The scale takes only a few minutes to complete. Scores for scale can range from 8 to 64, with higher scores representing higher hope levels. Studies reported by Snyder et al. (1991) showed that the ATHS has acceptable psychometric properties. For the total scale, internal consistency alphas ranged from 0.74–0.84.

### Subject selection

#### Recruitment of psychiatric patients

Patients who attended psychiatric clinics for follow up appointments, new cases, and emergency cases with the diagnosis of at least one of depressive disorder or anxiety disorder were approached to participate in this study. During the study period, a total of 971 patients were found to have at least one of depressive or anxiety disorders. Of the 971 patients, 243 were excluded, either due lack of language proficiency (not able to read either English or Bahasa Malaysia -the national language of Malaysia) or being unable to concentrate. Among those who qualified, 445 patients refused to participate without giving any specific reason. In the final count, 283 psychiatric patients participated in this study after giving their written consent. The Mini International Neuropsychiatric Interview (MINI: Sheehan et al., 1998) was administered on every 10th patient to confirm the diagnosis given by their psychiatrist. Patients took approximately 45 min to complete the entire set of questionnaires. Out of the 283 respondents, 168 completed the questionnaires in English and 115 in Bahasa Malaysia.

#### Recruitment of medical patients

Medical patients recruited in this study were from Medical clinics, Ear, Nose and Throat (ENT) clinics, Ophthalmology clinics and orthopedic clinics. A total of 247 patients were approached in these clinics while they were waiting for their consultation appointments. Out of the 247 patients, 47 were excluded; 27 due lack of language proficiency (not able to read either English or Bahasa Malaysia) and 20 due to psychiatric co-morbidity. In the final count, 200 medical patients participated in this study after giving their written consent. Patients took approximately 45 min to complete the entire set of questionnaires. Out of the 200 respondents, 93 completed the questionnaires in English and 107 in Bahasa Malaysia.

This study was approved by University Kebangsaan Medical Centre, Research Ethics Committee (Medical Research and Industry) and Behavioural and Social Sciences Ethical Review Committee (BSSERC), University of Queensland.

### Data analysis

Statistical Packages for Social Sciences (SPSS) version 20.0 and Analysis of Moment Structures (AMOS) version 20.0 software were used to analyse the data in this study. Internal consistency of items was evaluated with Cronbach's alpha coefficients. Correlations were used to analyze the construct validity of PANSI. The discriminative validity, specificity and sensitivity of the PANSI were analyzed using logistic regression analysis.

In confirmatory factor analysis (CFA), the model fit was tested using several indices. The model fit was deemed good when chi-square/*df* <3, Goodness-of-Fit Index (GFI), Adjusted–Goodness- of- Fit Index (AGFI), Comparative Fit Index (CFI) all >0.9, and Root Mean Square Error Approximation (RMSEA) <0.08 (Browne and Cudeck, 1993).

## Results

### Descriptive analyses

Among the total 483 participants in the study, 188 (39%) were males and 295(61%) were females. Among the psychiatric patients, 203(72%) were diagnosed with major depressive disorders, 65(23%) with co-morbid anxiety disorders and 15(5%) with co-morbid anxiety and depressive disorders. The participants' ages ranged from 16 to 75 years, with a mean of 42 years. In the sample, 127(26.2%) were single, 300(62.1%) were married, 27(5.6%) were divorced, 7(1.6%) were widowed (1.6%), 7(1.5%) were separated and, the marital status of 14(2.9%) was not known. Among the respondents, 258(53%) were Malays, 157(33%) were Chinese, 53(11%) were Indians and 15(3%) were from other ethnic groups. In the sample, 290(60%) had college or pre university level of education, 185(38%) had high school level of education, 9(1.8%) had only completed primary school, and 2(0.4%) were without any formal education.

### Confirmatory factor analysis

**Figure 1:**
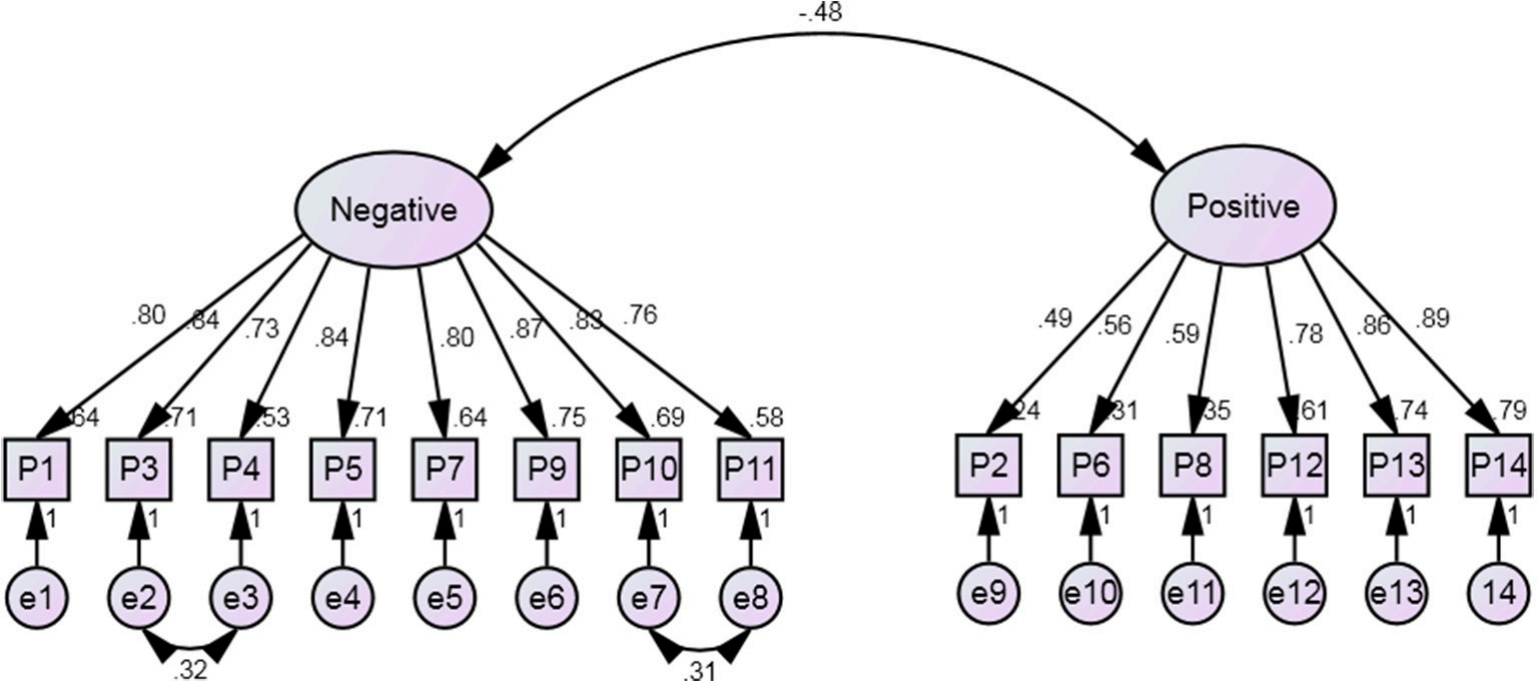
**Two factor PANSI measurement modelconstruct**.

After correcting for error terms, the two factor model, as shown in Figure 1, fitted very well (Chi-square/df = 2.451, GFI = 0.925, AGFI = 0.894, CFI = 0.963, RMSEA = 0.0680. The model fit was similar to that of Osman et al. (2002, 2003) and Muehlenkamp et al. (2005). The purpose of correlating item 3 “Felt hopeless and wondered” and item 4 “Felt unhappy about” was because these two items are quite close in what they measure. Hence, empirically the correlation between these items is slightly higher compared to other pairs. Therefore, to stabilize the model it is logical to correlate the two respective error terms (e2 and e3). Similarly for item 10 “Thought problems were overwhelming” and item 11 “Felt lonely,” the “thought problems” could be related to “lonely” because when the thought problems are overwhelming, one tends to feel hopeless and thus lost helpless. Thus, helplessness can be related to feeling of loneliness as no one cares.

The factor loadings for the PANSI-Negative items ranged from 0.73 to 0.87. The Average Variance Extracted (AVE) value, which is the average of the squared factor loading values, was 0.656. The factor loading for the PANSI-Positive ranged from 0.49 to 0.89 and AVE was 0.507. Hence, there is sufficient convergence validity of the items within the respective constructs. The correlation between the PANSI-Negative and PANSI-Positive latent constructs was −0.48. R-squared value of 0.230 was less than the Average Variance Extracted (AVE) values for PANSI-Positive and PANSI-Positive constructs. Hence, there is sufficient discriminant validity (Fornell and Larcker, 1981).

### Reliability analysis of the PANSI scales

The Cronbach's alpha values for the positive and negative scales were 0.93 and 0.84, respectively. The inter-item correlations ranged from 0.50 to 0.74 for PANSI positive and from 0.32 to 0.80 for PANSI negative. The value should be less than 0.85 (Kline, 2011). Hence, there was no problem with multicollinearity between the items in the constructs (Tabachnick and Fidell, 2007). The Cronbach's alpha values for RFL, RSE, ATHS, PSR, SWLS, and BHS were 0.94, 0.61, 0.76, 0.91, 0.86, and 0.78, respectively. For the domains in DASS21 the Cronbach's alpha values for depression, anxiety and stress were 0.92, 0.89, and 0.90, respectively.

### Construct validity

Table 1 presents the correlation matrix between PANSI and the other scales. All pairwise correlation values were statistically significant (*p* < 0.001). PANSI-positive is positively correlated with the five scales measuring protective factors and negatively correlated with the two scales measuring risk factors. PANSI-negative is positively correlated with the two scales measuring risk factors and negatively correlated with the five scales measuring protective factors. All the correlation values are moderate in magnitude. Hence, PANSI demonstrates good construct validity with the other scales.

**Table 1:** **Correlations of PANSI with other subscales**.

	**RFL**	**RSE**	**AHT**	**PSR**	**SWL**	**BHS**	**DASS**
PANSI positive	0.391	0.488	0.428	0.360	0.508	−0.519	−0.456
PANSI negative	−0.345	−0.355	−0.357	−0.398	−0.412	0.480	0.517

### Discriminative validity

Logistic regression analyses were used to evaluate the association between PANSI-negative and PANSI-positive scales and the patient status: either has attempted suicide (1) or had not attempted suicide (0). In both analyses, the reference group was those who did not attempt suicide. The results are shown in Table 2. There was a statistically significant association between PANSI-negative and patient status (estimate = 0.120, *p* < 0.05, OR = 1.128, 95% CI = 1.1, 1.2). This indicates positive relationship between PANSI-negative scores with the likelihood of suicidal attempt. The overall classification accuracy was 84.6%. There was a statistically significant association between PANSI-positive and patient status (estimate = −0.123, *p* < 0.05, OR = 0.884, 95% CI = 0.85, 0.92). The higher the PANSI-positive score, the lower odds of suicide attempt. The overall classification accuracy was 85.2%.

**Table 2:** **Results from logistic regressions**.

**Regression**	**Predictor**	***B***	***SE***	**Sig**	**OR(95% CI)**
I	PANSI-negative	0.120	0.017	<0.001	1.128 (1.091, 1.166)
	Constant	−3.396	0.293	<0.001	0.033
	PANSI-positive	−0.123	0.022	<0.001	0.884 (0.846, 0.924)
II	Constant	0.676	0.434	0.119	1.966

### Comparison of PANSI scores

Descriptive summary of PANSI-negative and PANSI-positive scores for psychiatric and medical patients are provided in Table 3. Based on independent samples *t*-tests, the psychiatric patients had lower PANSI-positive scores (*t* = 6.286, *df* = 481, *p* < 0.001) and higher PANSI-negative scores (*t* = 6.395, *df* = 481, *p* < 0.001) compared to medical patients. This showed that the PANSI has good criterion validity.

**Table 3:** **PANSI-negative and PANSI-positive scores for psychiatric and medical patients**.

**Patient**	**PANSI-positive** **(Mean ±*SD*)**	**PANSI-negative** **(Mean ±*SD*)**
Psychiatric patients	19.8 ± 5.92	13.5 ± 7.47
Medical patients	23.0 ± 5.15	9.80 ± 4.00

## Discussion

The purpose of the current study was to validate the factor structure and psychometric properties of the PANSI (Osman et al., 1998) in a sample of clinical outpatients in Malaysia. A two factor model was confirmed by the data. Items loading on PANSI-negative and PANSI-positive were consistent with Osman et al. (1998) model and results from other researchers (Osman et al., 2002, 2003; Muehlenkamp et al., 2005).

The PANSI proved to have sufficient internal consistency, with Cronbach's alpha values of 0.93 for PANSI positive and 0.84 for PANSI negative. Logistic regression showed that PANSI had good discriminative validity. PANSI-negative and positive as a risk measure was able to differentiate the patients between patients with suicide attempts and those without suicide attempts. This finding is consistent with results from the Osman et al. (2002) study. In addition, PANSI had good criterion validity whereby the mean score of PANSI negative was higher among psychiatric patients compared to medical patients. The mean score of PANSI positive was found to be higher among medical patients compared to psychiatric patients. The results of the present study further verified the construct validity of PANSI. Osman et al. (2002) also reported a positive relationship between PANSI negative and BHS and a negative relationship between PANSI positive and RFL in a sample of psychiatric adolescents.

This is one of the first studies to validate the PANSI for use in a culturally different population. This study provides clear evidence that the PANSI is reliable and a valid measure of suicidal ideation. The major strengths of the present study are the use of the CFA methodology and the direct application of a theoretically derived measure to a clinical setting and a specific sample.

In conclusion, PANSI appears to be a sound measure of risk and protective factors related to outpatient suicide related behaviors and it is comparable to other used self-report measures. With regards to this, PANSI can be used with confidence in the future, especially among outpatients.

### Conflict of interest statement

The authors declare that the research was conducted in the absence of any commercial or financial relationships that could be construed as a potential conflict of interest.
